# Prenatal Diagnosis of Isolated Agnathia-Otocephaly: A Case Report and Review of the Literature

**DOI:** 10.1155/2016/8512351

**Published:** 2016-08-04

**Authors:** Kazuhiro Kajiwara, Tomohiro Tanemoto, Chie Nagata, Aikou Okamoto

**Affiliations:** ^1^The Jikei University School of Medicine, Nishishimbashi 25-83-3, Minato-ku, Tokyo 105-8461, Japan; ^2^National Center for Child Health and Development, 2-10-1 Okura, Setagaya-ku, Tokyo 157-8535, Japan

## Abstract

Agnathia is a rare disease characterized by the absence of a mandible. Few cases of prenatally diagnosed isolated agnathia have been reported. We present a case report and review of the literature of prenatally diagnosed agnathia. A 38-year-old woman (gravida 0, para 0) was referred to our hospital at 28 weeks and 3 days of gestation for fetal evaluation because of polyhydramnios and suspected facial anomalies. Three-dimensional ultrasonography and MRI indicated agnathia. Premature rupture of the membranes occurred before the parents could reach a decision on the postnatal treatment. We performed emergency cesarean section on the second day of the 33rd week of gestation. The neonate was deemed nonresuscitable and he died of airway obstruction shortly after birth. Because agnathia is associated with very poor prognosis, accurate prenatal diagnosis and detailed counseling should be promptly provided before unexpected delivery to the parents for the determination of postnatal treatment.

## 1. Introduction

Agnathia-otocephaly is an extremely rare lethal anomaly characterized by absence of the mandible [1–4]. Prenatal diagnosis of agnathia is important for providing prompt counseling to the parents because most affected infants die soon after birth. However, a screening method for this condition has not been established. Furthermore, diagnosis tends to be delayed because of the absence of polyhydramnios in the second trimester. Here, we report a case of isolated agnathia that was diagnosed in the third trimester. Because of a delay incurred by the parents in making a decision for postnatal treatment, emergency cesarean section was necessary. Additionally, we conducted a literature review on the prenatal diagnosis of agnathia to shed light on the clinical course and characteristics of the condition.

## 2. Case Report

A 38-year-old woman (gravida 0, para 0) was referred to our hospital at 28 weeks and 3 days of gestation for fetal evaluation because of polyhydramnios and suspected facial anomalies revealed by ultrasound imaging. Her prenatal history was unremarkable. There was no family history of congenital anomalies, stillbirths, miscarriages, or consanguinity.

**Figure 1:**
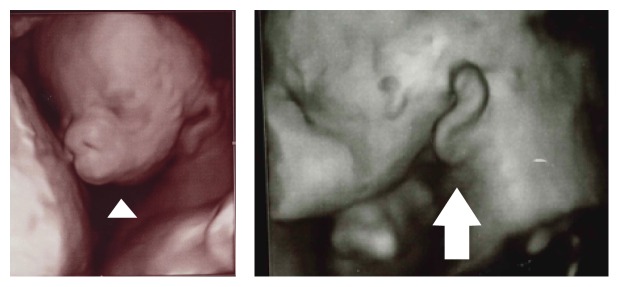
Three-dimensional ultrasonography demonstrated ventromedial displacement of the ear (arrow), small opening of the mouth, and mandibular agenesis (arrowhead).

**Figure 2:**
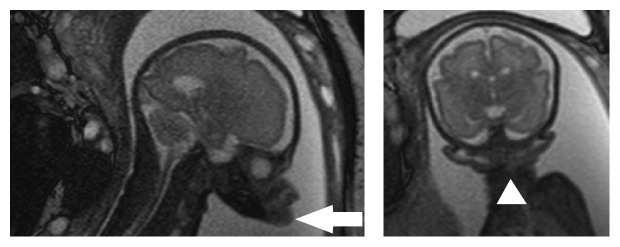
Magnetic resonance imaging demonstrated agnathia with low-set ventromedial displaced ear (arrowhead) and small opening of the mouth (arrow).

**Figure 3:**
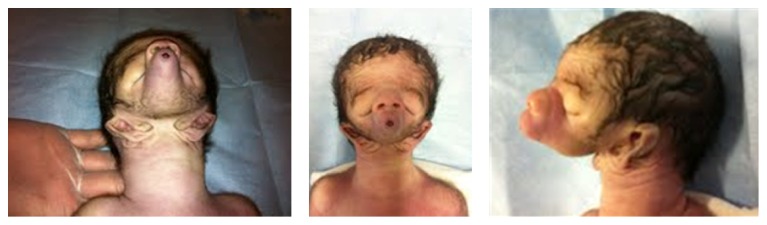
Postmortem image shows microstomia (characteristic of agnathia-otocephaly) and a proboscis with two nostrils.

At the initial visit, abdominal ultrasonography indicated an estimated fetal weight of 1,095 g, amniotic fluid index (AFI) of 32, and a small stomach bubble. Lower facial anomalies were assessed by three-dimensional (3D) ultrasonography, which revealed an absence of the mandible, low-set ventromedial displacement of the ear, and small opening of the mouth (Figure 1). We performed magnetic resonance imaging (MRI) to obtain additional findings, such as the ear position which was not clearly outlined by 3D ultrasonography due to the fetal position. MRI findings were consistent with the 3D ultrasonography findings and the ears were located in a much lower part of the face more than initially suspected by ultrasonographic findings (Figure 2). Lung position and lung volume appeared normal, and there were no ultrasonography and MRI findings which indicate secondary pulmonary hypoplasia. The amniotic fluid karyotype was 46, XY. We informed the parents that airway patency in neonate would be difficult to maintain and usually leads to death shortly after birth. We also provided the parents with the information regarding postnatal treatment options including active management, such as EXIT (*ex utero* intrapartum treatment), which may enable adequate airway management but may not be effective for pulmonary hypoplasia, as well as the preferable mode of delivery, which depends on their decision on the postnatal treatment. Before the parents could reach a final decision on the postnatal treatment, including EXIT, preterm premature rupture of membranes occurred at 33 weeks of gestation. We performed an emergency cesarean section. A male neonate with birth weight of 1,910 g was delivered. The appearance of the neonate suggested that he was nonresuscitable. The neonate died of total airway obstruction one hour after birth without active management. The neonate had isolated agnathia, microstomia, a proboscis with two nostrils, and downward-slanting palpebral fissures (Figure 3). The ears were low-set and nearly fused at the midline. Autopsy was not performed.

## 3. Discussion

The present case revealed a very important consideration; that is, we need to provide adequate information to the parents for them to make a prompt decision before delivery on the postnatal treatment agnathia. We conducted a literature review on agnathia and summarized its clinical course and diagnostic features. The information may contribute to the establishment of a screening method for this rare condition, as well as the timely treatment decision-making by the parents.

We searched the major electronic database PubMed using the search terms of “agnathia” and “otocephaly” and included all studies in English of prenatally diagnosed agnathia in human conducted between 1977 and 2016. The cases with unclear clinical course and micrognathia were excluded. The latest search was conducted on April 11, 2016. We reviewed all case reports of prenatally diagnosed agnathia. We extracted information on the diagnostic approach, gestational age at diagnosis, AFI, gestational age at delivery, associated anomalies, karyotypes, and outcomes.

Table 1 lists all the reported cases of prenatally diagnosed agnathia [5–26]. There were 22 cases of prenatally diagnosed agnathia. All cases, except one which was diagnosed during the first trimester (the 12th week) [23], were diagnosed after the second trimester. Facial screening during the first trimester has received much attention in recent years. Nonetheless, further improvement of the screening system is needed to reveal the characteristic facial features during the first trimester. Although several screening methods including inferior facial angle, jaw index, and mandibular ratio have been proposed, there is currently no definitive screening method for agnathia [27–29]. Instead, visualization of the mandible arch can be achieved by viewing the nuchal translucency in the first trimester as it provides visualization of frontomaxillary facial angle measurement [30]. In addition to routine facial screening, we recommend mandibular arch screening on the sagittal section of the face in the first or early second trimester, as this may guide clinicians to consider agnathia. During the third trimester, agnathia and polyhydramnios often occur together. However, in the first and early second trimester, polyhydramnios is often not observed. Hence, in early pregnancy, absence of polyhydramnios does not necessarily imply absence of agnathia (Table 1).

Three-dimensional ultrasound has been shown to be effective for the evaluation of malformed skull and face [31]. This leads to its use as the main tool for agnathia detection. Lee et al. reported its effectiveness in nine cases of micrognathia [32]. However, as pregnancy advances, visualization of the jaw may not be possible. Instead, helical CT and MRI may be more effective as fetal activity diminishes in late pregnancy. Furthermore, in some cases, the location of the fetal ears may be more evident in MRI than in 3D ultrasound. However, there is one major limitation of MRI. The imaging technique may not reveal the lower face depending on the position of the fetus in the uterus and the bend of the neck [11]. In view of that, helical CT images may be more effective as a prenatal diagnostic tool. Ebina et al. used helical CT to detect agnathia prenatally [7]. Compared with conventional CT, helical CT results in less radiation exposure (20–30 mGy) and image degradation due to motion artifact [7, 33].

Chromosomal tests were conducted in most of the reported cases. The tests indicated unbalanced translocation in some cases and the presence of two cases with trisomy 21, although the karyotype was normal in others [2, 34]. Additionally, micrognathia may occur with various chromosomal abnormalities. Therefore, chromosome test may be necessary in cases in which differentiation between agnathia and micrognathia is difficult. Agnathia usually occurs sporadically, but the involvement of genetic factors has been suggested based on two cases of recurrent agnathia [35, 36] and one case of a child with consanguineous parents [37]. In human agnathia, a heterozygous frameshift mutation in a possible causative gene* PRRX1* was identified [14, 26, 36–38]. Genetic testing using amniotic fluid cells may be useful for the prenatal diagnosis of agnathia. In a recently reported case of agnathia, the mother was found to consume alcohol regularly [39]. The teratogenic effects of prenatal alcoholic exposure may be exacerbated by Sonic Hedgehog haploinsufficiency and may in turn affect the development of the mandible [40].

Although the mode of delivery is largely dependent on the choice of postnatal treatment, the preferred method is cesarean section in cases of EXIT (*ex utero* intrapartum treatment) and breech presentation [11, 18]. However, the prognosis of agnathia is very poor even with adequate airway management, including EXIT, oropharyngeal intubation, and nasotracheal intubation [8, 11, 21]. Furthermore, absence of a passage between the trachea and pharynx or secondary pulmonary hypoplasia may render treatment more difficult. Nonetheless, a limited number of cases who survived more than one year have been reported [41–44].

In conclusion, when agnathia is suspected, it is important to provide sufficient counseling to the parents for them to make prompt treatment decision. The clinical course and characteristics of agnathia should be further studied to improve the accuracy of prenatal diagnosis of this condition.

## Figures and Tables

**Table 1:** Summary of case reports of prenatally diagnosed agnathia.

Author, year	Age (year)	Diagnostic approach	GA at initial diagnosis (week)	AFI	Karyotype	Major associated anomaly	GA at delivery (week)	Intervention after birth	Outcome	Survival period (day)
Scholl Jr., 1977 [15]	27	Radiograph ^*∗*^1	33	NA	Not performed	None	NA	/	NND	0
Dao et al., 1988 [16]	17	Ultrasound (2D)	32	NA	46, XY	None	NA	None	NND	0
Persutte et al., 1990 [17]	24	Ultrasound (2D)	20	NA	46, XX	MCA ^*∗*^2	21	/	IUFD	/
Brown and Marsh, 1990 [18]	23	Ultrasound (2D)	36	NA	46, XY	MCA ^*∗*^3	39	/	NND	5
Rolland et al., 1991 [5]	27	Ultrasound (2D)	23	Normal	46, XX	Polydactyly, holoprosencephaly	25	/	IUFD	/
Lin et al., 1998 [19]	30	Ultrasound (2D, 3D)	24	28.4	46, XX	None	24	/	TOP	/
Rahmani et al., 1998 [20]	33	Ultrasound (2D)	19	Normal	46, XY	None	NA	/	TOP	/
Ibba et al., 2000 [6]	32	Ultrasound (2D)	32	NA	46, XY	Hypoplastic thymus	32	None	NND	0
Ebina et al., 2001 [7]	36	Ultrasound (2D)/MRI/CT	22 + 2	19.5	46, XX	Holoprosencephaly, hemivertebrae, adhesion of the ribs	26 + 1	/	TOP	/
Yang et al., 2003 [8]	30	Ultrasound (2D)	31 + 2	45	46, XY	Hypospadia, cryptorchidism, subependymal cyst	32 + 1	Tracheostomy	NND	14
Chen et al., 2003 [9]	30	Ultrasound (2D)/MRI	29	45	46, XX	None	29	None	NND	0
Falcon et al., 2004 [10]	18	Ultrasound (2D)	18	Normal	46, XY	Tetraamelia, CDH, anal imperforation	NA	/	TOP	/
Umekawa et al., 2007 [11]	34	Ultrasound (3D)/MRI	26	47	46, XY	None	33 + 5	Tracheotomy (EXIT)	NND	3
Rajan et al., 2007 [21]	27	Ultrasound (2D, 3D)	32	54	46, XX	None	37	Tracheostomy	NND	0
Ducarme et al., 2007 [22]	35	Ultrasound (2D, 3D)	16	NA	46, XX	None	28	/	TOP	/
Chen et al., 2007 [23]	41	Ultrasound (2D), MRI	12	NA	46, XY	None	16	/	TOP	/
Tantbirojn et al., 2008 [24]	37	Ultrasound (2D, 3D)	26	Normal	46, XY	None	23	/	TOP	/
Huissoudc et al., 2008 [25]	37	Ultrasound (2D, 3D)	16	Normal	46, XY	None	17	/	TOP	/
Chaoui et al., 2011 [12]	NA	Ultrasound (2D, 3D)	Second trimester	Slightly increased	46, XY	Absence of the left kidney	NA	/	TOP	/
Donnelly et al., 2012 [26]	NA	Ultrasound (2D, 3D)	33	49	46, XX	None	33	Tracheostomy (failed)	NND	0
Akiyama et al., 2013 [13]	40	Ultrasound (2D, 3D), MRI	25	26	46, XY	None	38	None	NND	0
Patat et al., 2013 [14]	36	Ultrasound (2D)	28	Increased	46, XY	None	30	None	NND	0

^*∗*^1: radiograph taken after the injection of renografin.

^*∗*^2: anterior cervical cyst (hypopharynx), cyclopia, polysplenia, bilateral left lung, duodenal atresia, aplasia of the pituitary gland, adrenal hypoplasia, hydranencephaly, holoprosencephalic brain, absence of the internal carotid artery.

^*∗*^3: brachydactyly, syndactyly, micropenis with hypospadias and cryptorchidism, Dandy-Walker malformation, tetralogy of fallot, agenesis of the corpus callosum.
